# Survival and autoimmune risks post-thymectomy

**DOI:** 10.3389/fimmu.2025.1504496

**Published:** 2025-03-13

**Authors:** Irina Tsirkin, Mohamed Khateb, Dvir Aran, Amit Kaz, Shahar Shelly

**Affiliations:** ^1^ Department of Neurology, Assuta Medical Center, Ashdod, Israel; ^2^ Department of Neurology, Rambam Medical Center, Haifa, Israel; ^3^ Faculty of Biology, Technion-Israel Institute of Technology, Haifa, Israel; ^4^ The Taub Faculty of Computer Science, Technion-Israel Institute of Technology, Haifa, Israel; ^5^ Department of Thoracic Surgery, Rambam Medical Center, Haifa, Israel; ^6^ Neuroimmunology Laboratory, Ruth, and Bruce Rapaport Faculty of Medicine, Technion, Israel Institute of Technology, Haifa, Israel; ^7^ Department of Neurology, Mayo Clinic, Rochester, MN, United States

**Keywords:** thymic pathology, myasthenia gravis, thymectomy, mortality, autoimmune disease

## Abstract

**Background and objectives:**

Recent studies have raised concerns about thymectomy's deleterious effects. However, this conclusion was not exclusive to patients with myasthenia gravis (MG). The objective of this study was to test this hypothesis in thymectomy patients, regardless of their MG status.

**Methods:**

We conducted a retrospective case-control study to analyze clinical and radiological data from 1 January 2010 to 30 November 2023. Patients were divided into four groups: MG with (MG-Thy) or without thymectomy (MG-NO-Thy); thoracoscopic surgery without thymectomy (Surgery-NO-Thy) and Non-MG with thymectomy (Non-MG-Thy).

**Results:**

We identified a total of 456 patients (n=41, MG-Thy; n= 278, MG-NO-Thy; n=65, Non-MG-Thy; and n=72, Surgery-NO-Thy). The median ages were as follows: MG-Thy, 45.6 years (range: 22-79); MG-NO-Thy, 65 years (13-93); Non-MG-Thy, 59.8 (19-85) years; and Surgery-NO-Thy, 59.8 years (range: 19-85) (p<0.001). The median follow-up times were 5.5 years in MG-Tym, 3 in MG-NO-Thy, 3.9 in Non-MG-Thy, and 4.7 years in Surgery-NO-Thy. A thymic mass was detected with chest computed tomography (CT) in 56% (23/41) of the MG-Thy cohort and in all the Non-MG-Thy cohort. Thymic pathology in the MG-Thy group showed normal/fat atrophic thymus in 31.7% (13/41), hyperplasia in 26.8% (11/41), thymic cyst in 2.4% (1/41), and malignant in 39% (16/41). Thymic pathology in the non-MG group showed hyperplasia, fat, or normal thymus in 16.9% (11/65); thymic cyst in 18.5% (12/65); malignant thymoma in 60% (39/65); and others in 4.6% (3/65). The death rate was the lowest in the MG-Thy group, compared to the non-MG groups and the MG-No-Thy group. Specifically, death occurred in zero cases in the MG-Thy group, while it occurred in 13.8% (9/65) of the thymectomized non-MG group and in 35.6% (99/278) of the MG-without thymectomy group. Excluding late-onset MG patients (LOMG), the death incidence was 14.4% (15/104). The prevalence of autoimmune diseases before thymectomy was 14.6% (6/41) in the MG-Thy group versus 12.3% (8/65) in the Non-MG-Thy group, with three new cases post thymectomy in non-MG group. Post thymectomy cancer incidence was zero in the MG-Thy group, versus 16.2% (45/278) in the MG-NO-Thy group.

**Conclusion:**

The benefits of thymectomy outweigh potential risks for patients with MG or patients with thymic malignancies. Incidental thymectomy should be avoided. This call for reevaluation of thymectomy especially for non-neoplastic causes.

## Introduction

The thymus plays a crucial role in the development of the immune system. Specifically, it is essential for the formation of a functional T-cell repertoire (1). Historically, the thymus has been recognized as a crucial hematopoietic site for T cell development and maturation of the adaptive immune system (2, 3). This understanding has evolved from the earlier belief in its limited participation in immune reactions, highlighted by experiments in the 1960s that demonstrated the thymus’s essential role in immunological response, particularly in early life stages (4). Such work laid the foundation for appreciating the thymus’s complex contributions to immune homeostasis, including its involvement in T lymphocyte differentiation, the selection process for T cells that recognize foreign antigens, and the elimination of self-reactive T cells (3). However, its role in adults is not well understood (5, 6).

Thymomas and thymic carcinomas comprise thymic epithelial tumors with an estimated incidence rate of approximately 0.22 per 100,000 annually (7). Thymectomy was shown to be a beneficial therapeutic intervention in early-onset seropositive (Ach receptor) myasthenia gravis (MG) patients, especially with the existence of thymoma (8). Recent evidence from a comprehensive study conducted by Kooshesh et al. in patients with thymic removal has ignited fresh concerns regarding its safety and long-term effects (9). The study examined outcomes for 1,420 patients who had undergone thymectomy compared to 6,021 controls. The primary cohort included 1,146 thymectomy patients with matched controls. At 5 years post-surgery, the thymectomy group had higher all-cause mortality [8.1% vs. 2.8%; relative risk (RR), 2.9] and a higher risk of cancer (7.4% vs. 3.7%; RR, 2.0). Although the overall risk of autoimmune disease was similar between groups, excluding patients with preoperative conditions revealed a higher risk in the thymectomy group (12.3% vs. 7.9%; RR, 1.5). Higher all-cause and cancer mortality were found in the thymectomy group compared to the general U.S. population. This pivotal research highlights the critical interaction between thymectomy and subsequent health outcomes. However, extrapolating from the conclusions of this study in MG patients may be problematic because most of their cohort was non-myasthenic.

Another study aimed to examine the long-term effects of complete thymectomy performed early in life on autoimmunity and allergy. Comparing adults who had undergone thymectomy in infancy with age-matched healthy controls (10), the study found no significant increase in autoimmune manifestations or allergy symptoms. Adults who had complete thymectomy early in infancy showed a substantial reduction in naïve CD4+ and CD8+. However, the regulatory T-cell compartment, marked by FoxP3, CTLA-4, and CD39, was preserved, suggesting thymectomy did not significantly impair the suppressive functions of Tregs. Roosen et al. conducted a study on the impact of routine thymectomy during congenital cardiac surgery on adaptive immunity and its clinical implications. They did show changes in adaptive immunity, however, the study found no significant clinical consequences. There was no increase in the incidence of infections or autoimmune diseases, suggesting that the immune system can compensate for the loss of the thymus to some extent (11).

Herein, we evaluated the long-term outcomes of thymectomy in patients with MG compared to three different groups: patients without an MG diagnosis, patients with an MG diagnosis, and patients who had thoracoscopic surgery alone with no thymic removal or pathology. Specifically, we aimed to assess the impact of thymectomy on overall mortality, the incidence of extrathymic cancers and autoimmune diseases, and surgery-associated complications.

## Methods

### Cohort selection

**Figure 1:**
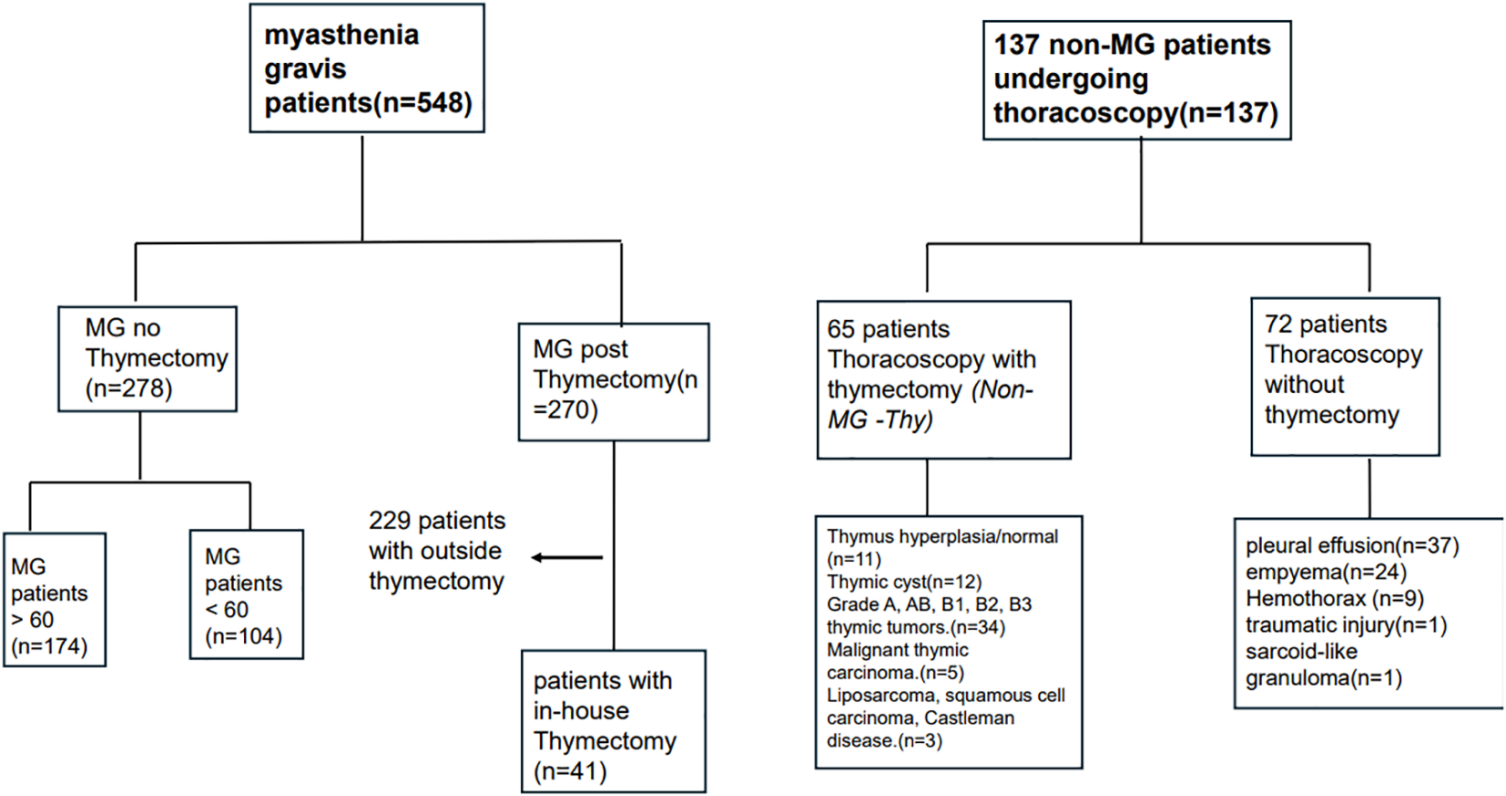
Flow chart for study design.

The Rambam Medical Center (RMC) institutional review board ethical committee approved this study. We included four groups of patients: MG with thymectomy (MG-Thy), MG without thymectomy (MG-NO-Thy); thoracoscopic surgery without thymectomy (Surgery-NO-Thy); and Non-MG with thymectomy (Non-MG-Thy) (please see **Figure 1**). We retrospectively reviewed patients with MG (with or without thymectomy) who were referred to our neurological department or clinics in our tertiary center. For the patients with thymectomy and thoracoscopy without thymectomy, we extracted data from the records of the thoracic surgery department and clinics at our tertiary institution.

### MG with and without thymectomy (MG-Thy, MG-NO-Thy)

In the MG cohorts, we confirmed the diagnosis of myasthenia at our institution and selected patients seen between 1 January 2010 and 30 November 2023. They were then classified into patients who had undergone thymectomy and no thymectomy (MG-Thy and MG-NO-Thy). The Non-MG-Thy group were patients who had undergone thymic removal without a diagnosis of MG. They did not have any complaints of weakness at any time pre-surgery and all were reviewed by an expert neurologist. Finally, the fourth group consisted of patients who only had thoracoscopic surgery (Surgery-NO-Thy). None had malignancy, a diagnosis necessitating ICU, or any other surgical intervention other than thoracoscopic surgery. All had their whole thymus seen.

MG diagnosis in our study combines a clinical impression of MG and supportive electrodiagnostic (EDX) testing or positive antibody testing reported or reviewed by our team (12). Exclusion criteria were (1) immune modulatory therapy 6 months before antibody testing or (2) use of acetylcholine esterase inhibitors within 8 hours of electrodiagnostic (EDX) testing. Repetitive nerve stimulation (RNS) was considered confirmatory when a pathologic pattern of decrement of >10% was seen in the compound muscle action potential (CMAP) at baseline or up to 3 minutes post 1-minute exercise in two or more motor nerves. Stimulatory single fiber electromyogram (SFEMG) was also performed in suspected patients. SFEMG positivity was determined by utilizing the quality and cutoff guidelines previously published and after excluding alternative diagnoses such as myopathy, neuropathy, or motor neuron disease (13). MG patients were followed up within our neuromuscular clinic. They were evaluated by an expert neurologist (M.K or SH.SH) at each visit. Average follow-up time was 4.8 years (range:1-10 years) and 4.89 years (range:1-33 years) for MG patients with and without thymectomy, respectively. The follow-up of MG patients is clinical and does not include routine electrophysiological or serological follow-up. These tests, although substantial at the diagnosis, were only occasionally performed during the follow-up time and only for specific reasons, such as to prove remission.

We included all clinical and radiological data that were collected from the electronic medical system. A comprehensive evaluation of each case was undertaken for the chart review process, ensuring accuracy (S.S and I.S). Patients who had thymectomy done outside of our institution were excluded from this study. The MG without thymectomy (MG-NO-Thy) cohort consisted of 278 patients. All the included patients, except for the control group of MG without thymectomy (MG-NO-Thy), must have had at least one neurological or chest surgery medical visit a year post-operation or more. The thymectomy was performed based on the established MGTX criteria, including generalized MG, Ach-R-Ab positivity, and disease duration of less than 2 years in cases with no thymic mass. However, there were cases where thymectomy was conducted for patients who did not meet the previous criteria but had refractory MG (after 5^th^ line of treatment) or patients with MG and suspicious findings for thymoma. In such instances, the decision to proceed with surgery was made following multidisciplinary consensus, ensuring a comprehensive evaluation of clinical indications and patient-specific factors. Pre- and post-treatment indicators, variations in treatment, and the number of changes in clinical status according to the Myasthenia Gravis Foundation of America (MGFA) were used to determine the effect of thymectomy (13, 14).

In the MG-NO-Thy group, we further improved the reliability of this group by creating a subgroup of MG-NO-Thy that was matched according to age and sex with the MG-Thy group. Age-and-sex matching was achieved by the “nearest neighbor” method. After matching, this group had 101 patients (**Table 1**).

### Thymectomy without MG group (Non-MG Thy)

We reviewed our electronic medical records to identify all thymectomy procedures performed at our institution within the specified timeframe. Eligible patients were those with available chest computed tomography (CT) imaging, pathological diagnoses, and follow-up data at our institution. Patients presenting with weakness or muscle-related symptoms during their initial surgical consultation were excluded from the analysis.

### Thoracoscopy cohort (Surgery-NO-Thy)

We reviewed our medical records to identify all patients who underwent thoracoscopic procedures excluding patients with a malignancy diagnosis necessitating surgery, patients hospitalized in the ICU, or any other surgical intervention other than thoracoscopic surgery. All had thymic tissue in chest imaging.

This group was added to mitigate any effects related to the procedure itself. Thoracoscopic procedures, as with any invasive surgical intervention, may have inherent risks that could influence outcomes such as mortality. Including this group allowed us to better isolate the impact of thymectomy from procedure-related factors, which could otherwise confound the results.

### Endpoints and statistical considerations

Endpoints were death from any cause, cancer development with histopathological confirmation, and development of autoimmune disease. Group differences were established using the Wilcoxon rank-sum test between the means of continuous variables, and the Chi-squared test for the categorical variables. Survival was analyzed using Kaplan–Meier curves and Cox regression analysis. P < 0.05 was regarded as statistically significant.

### Full data access statements

The authors take full responsibility for the data, the analyses and interpretation, and the conduct of the research. They have full access to all the data, and they have the right to publish all data. Anonymized data is not published within this article and will be made available by request from any qualified investigator.

## Results

### Thymectomized myasthenia cohort (MG-Thy)

We identified 41 patients with a confirmed MG diagnosis who had thymectomy at our institution and met all inclusion criteria. The median age at diagnosis was 45.6 (range: 22-79 years) for men and 44.9 (range: 15-82 years) for women. MG symptoms were recorded as ocular (14.6%, 6/41) or generalized (85.3%, 35/41; **Tables 1**, **2**). The median follow-up time was 5.5 years with a mean of 4.8 years (range:1-10 years). Serological status was available for review in 40/41 patients, with 77.5% seropositive (31/40, 77.5% for AchR and none were positive for anti-MuSK) and the remaining 22.5% (9/40) seronegative (**Table 1**). In the seronegative MG patients, thymectomy was performed due to a thymic mass (n=9) on CT. Chest CT was performed in 100% (41/41) with thymic mass detected in 56% (23/41). Thymic removal was carried out in all patients as per inclusion criteria, 39% (16/41) were a histopathologically malignant grade and 26.8% (11/41) of cases had hyperplasia. There was a normal or fat atrophic thymus in 31.7% (13/41), and a thymic cyst in 2.4% (1/41). Time to thymic removal from symptoms onset was on average 1.5 years (range: 0–8 years). Follow-up time post thymic removal was 3.7 years (range 1–10 years).

**Table 1:** MG-related parameters.

Variable	MG-Thy (n=41)*	MG-NO-Thy (n= 278)*	MG-NO-Thy (excluding those >60 years old, n=104)	Matched MG-NO-Thy (excluding those >60 years old, and with age and sex matching with the first group, n=101)	P-value
Age median (range, years)	45.6 (22-79)	65.5 (13-93)	46 (13-60)	46 (13-60)	P<0.001 P=0.501 between the MG-Thy and the age-and-sex-matched MG-NO-Thy groups.
Follow-up time (average in years)	4.8	4.89	5.97	5.85	P=0.13
Females (%)	70.7%	46.8%	54.8%	56.4%	P=0.12 between the MG-Thy and the age-and-sex-matched MG-NO-Thy groups.
MG presentation	14.6%- ocular. 85.3%- generalized.	65.5%- ocular.34.5%- generalized.	67.3%- ocular.32.7%- generalized	67.3%- ocular.32.7%- generalized	P < 0.05 for the ocular and generalized ratio, between the first and each of the other columns. P= 0.3 and 0.73 for the ocular and generalized ratio between the second and third columns.
Ach-R Ab seropositive	77.5%	71.3%	56.4%	58.4%	NA
Steroids	61%	NA	NA	53%	P= 0.42
Azathiopirine	63%	NA	NA	36%	p<0.01
Chronic IVIG**	39%	NA	NA	13%	p<0.01
Chronic PLEX**	42%	NA	NA	19%	p<0.01
Novel biological treatments (rituximab and eculizumab)	27%	NA	NA	8%	p<0.01
Mestinon only	10%	NA	NA	36%	p<0.01
Cancer post thymectomy/post MG diagnosis	None	16.2% (45/278)	14.4% (15/104)	14.85% (15/101)	P<0.001
Mortality	None	35.6% (99/278)	14.4%, (15/104)	13.86% (14/101)	P<0.001

Demographic features, cancer incidence, mortality, clinical, serological, and treatment comparisons between the different groups with MG.

*MG-Thy, Myasthenia Gravis with thymectomy.

*MG-NO-Thy, Myasthenia Gravis without thymectomy.

**IVIG, intra venous immunoglobulins.

**PLEX, Plasma exchange/pheresis.

NA, Not applicable.

**Table 2:** Demographics and thymic pathology.

Variable	MG-Thy (n=41)*	MG-NO-Thy (n= 278)*	Non-MG-Thy (n=65) *	Surgery-NO-Thy (n=72)*	P-value
Age median (range, years)	45.6 (22-79)	65.5 (13-93)	59.8 (19-85)	55. 5 (22-82)	P<0.001 P=0.501 between the MG-Thy and the age-and-sex-matched MG-NO-Thy groups.
Follow-up time (average in years)	4.8	4.89	3.9	4.7	P=0.13
Females (%)	70.7%	46.8%	50.8%	14%	P=0.12 between the MG-Thy and the age-and-sex-matched MG-NO-Thy groups.
Pathological classification (malignant thymic)	39%	NA	63.1%	NA	NA
Benign pathology of the thymus	61%	NA	35.4%	NA	NA
Cancer post thymectomy/post MG diagnosis	None	16.2% (45/278)	27.7% (18/65) 6.2% (4/65) two or more cancer types	NA	P<0.001
Mortality	None	35.6% (99/278)	13.8% (9/65)	11% (8/72)	P<0.001
Autoimmune diseases pre-thymectomy	14.6% (6/41)	NA	12.3% (8/65)	NA	NA
Autoimmune diseases post-thymectomy	0	NA	4.6% (3/65)	NA	P=0.16

General demographic features, thymic pathology, cancer and autoimmune incidence, and mortality, in each of the groups.

*MG-Thy, Myasthenia Gravis with thymectomy.

*MG-NO-Thy, Myasthenia Gravis without thymectomy.

*Non-MG-Thy, Thymectomized patients without Myasthenia Gravis.

*Surgery-NO-Thy, Thoracoscopy without thymectomy.

NA, Not applicable.

### Thymectomized non-MG cohort (Non-MG-Thy)

We identified 65 patients who underwent thymic removal between 1 January 2010 and 31 December 2023 at our institution, with available pathological and follow-up data. Median age was 59.8 (range 19-85 years) at the time of thymic removal and 49.2% were men and 50.8% were women. Causes leading to the discovery of the thymic mass included incidental imaging due to chest trauma or weight loss in 54%, chest pain in 15%, dyspnea in 12%, cough and hemoptysis in 11%, pneumonia in 6%, and non-specific visual complaints in 2%. The median follow-up time was 3.9 years (range 1–13). All the thymic epithelial tumors were classified according to the WHO histologic classification and the Masaoka clinical staging system (7). Pathology data was available in all patients, showing 16.9% (11/65) had thymic hyperplasia or normal thymus, 18.5% (12/65) had thymic cysts, 52.3% (34/65) had grades A, AB, B1, B2, and B3 staged thymic tumors, 7.7% (5/65) had malignant thymic carcinoma, 1.5% (1/65) had liposarcoma, and 1.5% (1/65) had necrotizing squamous carcinoma. One patient was diagnosed with Castleman disease (1.5%).

### Thoracoscopy cohort (Surgery-NO-Thy)

We identified 72 patients who had thoracoscopic exploration without cancer. Indications for decortication were pleural effusion in 51.4% (37/72), empyema in 33.3% (24/72), hemothorax in 12.5% (9/72), traumatic injury in 1.4% (1/72), and sarcoid-like granulomas in 1.4% (1/72). The median age was 55.5 years (range: 22 to 82), and eight patients died during the follow-up period.

### MG without thymectomy cohort (MG-NO-Thy)

We identified a cohort of 278 MG patients who did not undergo thymectomy. The median age at diagnosis was 66 (range: 17–93 years) for men and 64 (range: 13–86 years) for women. The median follow-up time was 3 years with a mean of 4.89 years (range:1–33 years). MG presentation was recorded as ocular (65.5%, 182/278) or generalized (34.5%, 96/278). Of the patients with available serological status, 71.3% (92/129) were seropositive for anti Ach receptor antibody, 3.9% (5/129) were seropositive for anti-MUSK antibody, and the rest (24.8%, 32/129) were double seronegative. Of these 278 MG patients who did not have thymectomy, 37.4% (104/278) were diagnosed at the age of 60 or before. Follow-up time was similar between the groups (p=0.13, **Tables 1**, **2**). We further improved the reliability of this group by matching according to age and sex with the MG-Thy group. After matching this group, we had 101 patients with a median age of onset of 46 years (13-60), a mean follow-up time of 5.85 years, and 56.4% were women (p>0.05 for age and sex compared to the MG-Thy group, **Table 2**).

### Cancer incidence

Malignancy was not reported during the follow-up period in any of the MG with thymectomy cohort. In the MG without thymectomy group, we identified a 16.2% (45/278) incidence of extrathymic cancers. When considering only the subgroup of patients diagnosed with MG at the age of 60 or before, this incidence was equal to 14.4% (15/104), which is dramatically higher than that in the group of MG with thymectomy (zero). The distribution of cancer in this group was as follows: dermatological, 5; hematological, 3; genital/gynecological, 3; renal/urinary, 1; gastrointestinal, 1; thyroid, 1; and lung, 1. Malignancy was reported in three cases, at the pre-thymectomy time, in the Non-MG Thy group. Cancers included acute myeloid leukemia, prostate carcinoma, and thyroid papillary carcinoma. Post thymic removal, malignancy was more common in the non-MG cohort with 18 distinct cases diagnosed post thymectomy, versus none in the MG with thymectomy cohort (p<0.001), with four cases having >1 primary malignancy. Thymic pathology was categorized into four main groups: normal/atrophied/cystic thymus, hyperplastic thymus, non-invasive thymoma, and invasive/malignant thymoma. The majority of the post-thymectomy cancers (78%, 14/18) were found in the last two groups, with an equal distribution (38.9%, 7/18) in both the non-invasive and invasive thymoma groups, and 22.2% (4/18) in the normal/atrophied/cystic thymus group. No cancers were observed in the hyperplastic thymus group.

### Autoimmune disease incidence

Autoimmune diseases were reported in six patients in the MG-Thy group including systemic lupus erythematous (n=3), Hashimoto’s thyroiditis (n=2), and rheumatoid arthritis (n=1) versus eight cases in the non-MG thymectomy group (p=0.73). Post thymectomy, three non-MG patients developed new autoimmune disease versus none in the MG-Thy cohort, which did not result in clinical significance (p=0.16). The three post-thymic autoimmune disorders in the Non-MG-Thy group occurred with a normal, atrophied, or cystic thymus, respectively.

### Mortality

**Figure 2:**
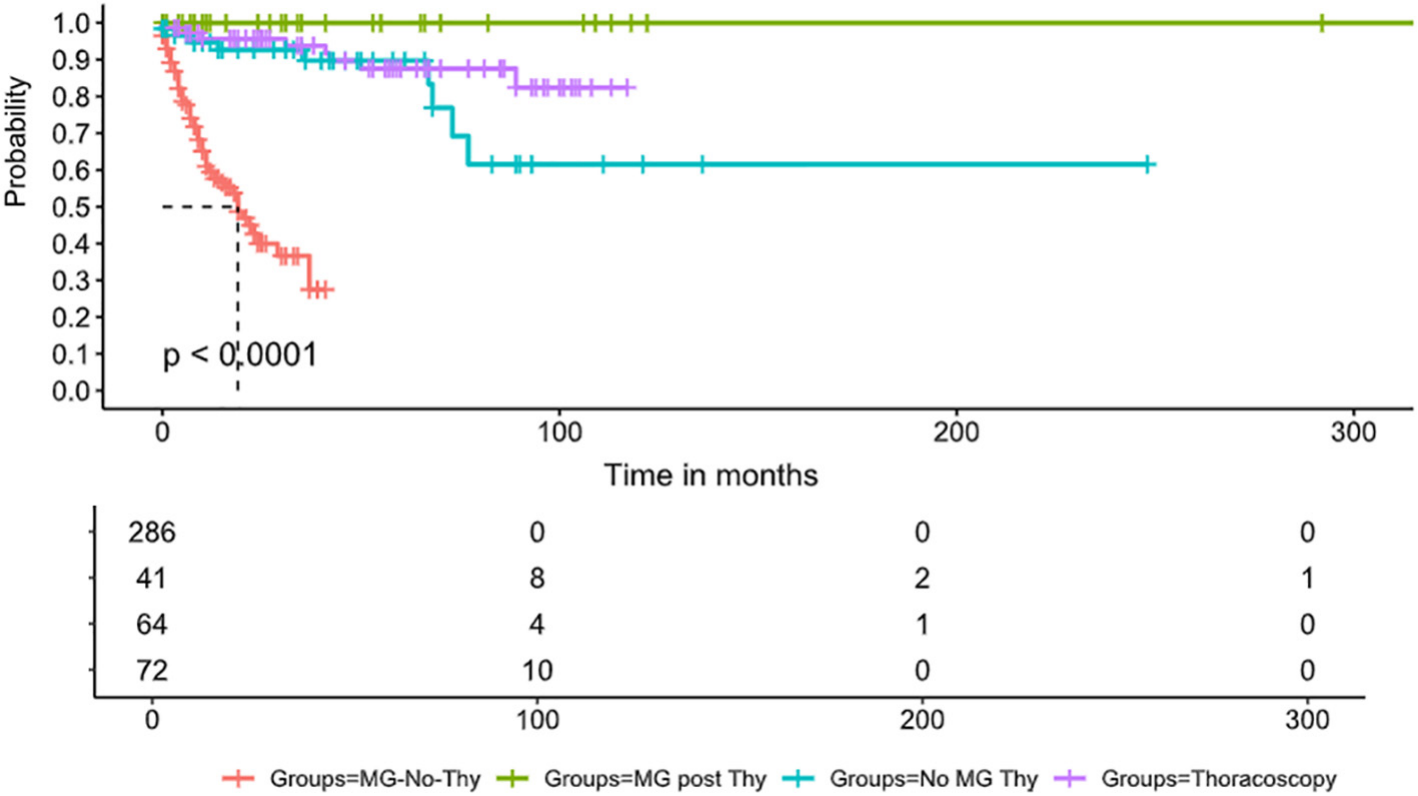
Survival comparison. Survival curve displays the probability of survival over time (in months) in four groups of patients: the red line denotes MG patients who did not undergo thymectomy, the blue line denotes MG patients who underwent thymectomy, the orange line denotes non-MG patients who underwent thymectomy, and, finally, the green line denotes patients who underwent thoracoscopy without thymectomy. The survival curves reveal a significant difference in survival probabilities between the groups (p < 0.0001). The MG-No-Thy group exhibits substantially lower survival rates, emphasizing increased mortality in this cohort. In contrast, the MG-post-Thy group shows the highest survival probability. The table below the plot provides the number of patients at risk at different time points in each group.

Mortality data was available for all the cases. The death rate was the lowest in the MG-Thy group, compared to the non-MG groups and the MG-No-Thy group. Specifically, death occurred in zero cases in the MG-Thy group, while death occurred in 13.8% (9/65) of the cases in the thymectomized non-MG group and in 35.6% (99/278) of the cases in the MG-without thymectomy group. Even after age and sex matching between this group and the MG-Thy group, the death incidence was still higher (13.86%, 14/101; p<0.001). In the non-MG group, the median time from thymectomy to death was 36 months post-surgery (range: <1 to 77 months). In the patients who died after thymectomy, thymic pathology included thymic carcinoma (n=3), thymoma type AB (n=2), thymoma B2 (n=1), thymoma B3 (n=1), squamous cell cancer (n=1), and thymic cyst (n=1). One non-MG patient had two thymectomies, the first in 1989 and the second in 2013 due to recurrence, and died within 2 months after surgery. The median age at death was 71.7 years in the non-MG group (range: 47–89 years), 73.6 years for men (range: 62–89 years), and 70.6 (47–84 years) for women. To assess the death risk in patients who had thoracoscopic surgery, we included another control cohort of patients who underwent thoracoscopic surgery in which the thymus remained intact. We compared mortality to show higher mortality rates in patients who underwent thymectomy (p=0.04) (**Figure 2**).

## Discussion

Incorporating the historical and recent findings on thymectomy’s role in immune system modulation, our study aimed to describe the clinical outcomes and implications of thymic removal. Recent emerging data suggest a paradoxical outcome of reduced survival and an increased incidence of autoimmune diseases following thymectomy (9), raising concerns over its long-standing position as a beneficial intervention in certain pathologies. Our study corroborates the recent findings regarding thymic removal in patients without MG, indicating adverse long-term outcomes and elevated mortality rates. Conversely, within the MG cohort, in which we have recently showed an increased incidence of extrathymic cancers (12), thymectomy was not linked to higher mortality rates or increased prevalences of cancer or autoimmune diseases when compared to the surgical control groups. These findings align with and expand upon the results of the MGTX trial, which provided robust evidence supporting the role of thymectomy in MG, particularly for patients with generalized acetylcholine receptor antibody-positive disease (8). The trial demonstrated that thymectomy, in conjunction with prednisone, significantly improved clinical outcomes, reducing disease severity and corticosteroid dependency compared to medical therapy alone (8). A possible explanation for our findings is that thymic removal depends upon its role in the disease. In MG patients, removing the abnormal thymus improves the condition by reducing exacerbations, hospitalizations, intubations, and mortality. It also decreases reliance on steroids and immunosuppressants. However, in those without MG, removing a normal thymus might cause immune imbalance, leading to more autoimmune diseases and cancers, and thus higher mortality.

We hypothesize that thymectomy, particularly in adult patients, may precipitate a cascade of immune dysregulation. This hypothesis was supported by the observed increase in post-thymic autoimmune diseases, cancer incidence, and mortality rates in our thymectomized non-MG cohort. Removal of the thymus—integral for T-cell selection and central tolerance—can lead to a decrease in immune regulatory capacity and an increased vulnerability to autoimmune and possibly other immunopathological conditions (9, 15). Our hypothesis is supported by recent retrospective research involving 445 patients with MG that was completed in Beijing Hospital by Tian et al. and which explored the effect of thymectomy on developing an autoimmune disease. Among the patients enrolled, 398 were not diagnosed with autoimmune disease at the time of the operation. Subsequently, after a median follow-up period of 72 months, 19/398 (4.8%) developed an autoimmune disease, in which Hashimoto’s thyroiditis and rheumatoid arthritis were the most prevalent. A literature review and four case reports suggest a potential correlation between thymectomy and systemic lupus erythematosus (SLE) development; this study found that 16 patients, most of whom were women, developed SLE after the procedure, and half of the cases occurred after 3 years post-surgery (16). In the most recent and comprehensive review of cases of thymoma-associated SLE, Jamilloux et al. reviewed 51 cases, finding that 84% were women, with 42% occurring post-thymectomy (17). Our findings, along with previous studies, suggest thymus removal should only be done with clear indications, such as a thymic mass in imaging or to improve MG patient outcomes. There is sufficient data to support removing the thymus only when necessary. Autoimmune diseases may take time to develop after thymectomy. Long-term surveillance strategies should include routine clinical follow-ups and patient-reported data, such as the Connective Tissue Disease Screening Questionnaire (CSQ) (18). Laboratory tests such as complete blood count (CBC), anti-nuclear antibody (ANA), and C-reactive protein (CRP) may be considered.

We did not observe a reduced survival rate among MG patients who underwent thymectomy compared to those who did not, indicating a potentially favorable outcome from thymectomy for MG, which is also supported by other studies (8, 14). However, the broader implications of thymectomy become evident when considering the significant mortality rate observed in the thymectomized non-MG group (13.8%). These findings echo the landmark study by Kooshesh et al., which identified an increased risk of cancer, autoimmune diseases, and death from any cause in adult thymectomy patients. Such outcomes highlight the critical balance disrupted by thymectomy, underscoring the thymus’s role in maintaining immune homeostasis and the potential consequences of its removal (19–21).

Our study has several limitations. It is retrospective with a small sample size. The thymectomized MG group was younger than the control groups, potentially affecting results. We found similar results in MG patients diagnosed at 60 years old or younger without thymectomy. In addition, in few cases there were deviations from MGTX criteria and differences in thymic pathologies between the groups. Other limitations include the effects of immunosuppressive treatments, unmitigated comorbidities in MG patients, and a lack of preoperative autoimmune screenings for subclinical conditions.

In summary, our study highlights the importance and complexity of thymic tissue in adult immune regulation, reinforcing the significant long-term effects associated with thymectomy in both MG and non-MG patients. Our findings support and augment previous research, particularly the review by Kaminski et al. (22) For MG patients, our data support the established benefits of thymectomy, as corroborated by randomized clinical trials and long-term studies. These findings demonstrate a reduction in mortality, lower rates of extrathymic cancer, and improved disease outcomes, particularly in patients with acetylcholine receptor antibody-positive generalized MG. This supports current clinical guidelines recommending thymectomy for MG patients under specific conditions.

However, the data also reveal that thymectomy in non-MG patients may be associated with increased risks of mortality, cancer, and autoimmune diseases, as highlighted in the concerns raised by Kaminski et al. This observation calls for a reevaluation of thymectomy in treatment plans, especially for non-malignant cases or incidental thymic removal during unrelated thoracic surgeries. The findings emphasize that thymectomy should be performed only with specific and clear indications, such as the presence of a thymic mass on imaging or to improve clinical outcomes for MG patients. Taken together, our results, combined with existing literature, suggest that unnecessary removal of the thymus should be avoided to minimize long-term risks. This observation calls for reevaluation of themetomy especially for non-neoplastic cases or incidental thymic removal during unrelated thoracic surgeries.

## Data Availability

The original contributions presented in the study are included in the article/supplementary material. Further inquiries can be directed to the corresponding author.
